# Supplementary material for: Optimizing glycine concentration to enhance gibbsite-catalyzed abiotic humification of catechol and glucose
Source: PLoS One. 2025 Nov 18;20(11):e0335528. doi: 10.1371/journal.pone.0335528 (PMC12626293; doi:10.1371/journal.pone.0335528)
Supplement: S1 Table — (DOCX) [file pone.0335528.s001.docx]

**S1 Table** *E*_4_/*E*_6_ ratios and SD values of supernatant at different culture times

| Treatments | 0 h | 3 h | 6 h | 18 h | 28 h | 48 h | 76 h | 124 h | 240 h | 360 h |
| --- | --- | --- | --- | --- | --- | --- | --- | --- | --- | --- |
| Gly0 | 4.71±0.24 | 2.30±0.16 | 2.20±0.26 | 1.42±0.52 | 1.39±0.15 | 1.43±0.16 | 1.43±0.16 | 1.57±0.17 | 1.64±0.18 | 1.62±0.18 |
| Gly0.03 | 3.78±0.21 | 1.90±0.18 | 1.61±0.17 | 1.57±0.42 | 1.69±0.19 | 1.65±0.18 | 1.63±0.18 | 1.75±0.19 | 1.56±0.17 | 1.87±0.21 |
| Gly0.06 | 3.98±0.44 | 3.30±0.22 | 2.01±0.21 | 1.92±0.37 | 2.16±0.24 | 1.97±0.22 | 1.98±0.22 | 2.07±0.23 | 1.67±0.18 | 1.80±0.23 |
| Gly0.12 | 6.33±0.36 | 5.20±0.24 | 2.58±0.25 | 2.27±0.27 | 2.42±0.27 | 2.47±0.27 | 2.52±0.28 | 2.55±0.28 | 2.18±0.29 | 2.11±0.04 |
| Gly0.24 | 4.05±0.29 | 2.65±0.24 | 2.13±0.30 | 2.73±0.13 | 2.49±0.21 | 2.74±0.15 | 3.04±0.15 | 3.08±0.23 | 3.70±0.16 | 3.77±0.12 |
| CK | 0.60±0.07 | 1.25±0.14 | 1.15±0.13 | 1.50±0.17 | 1.38±0.15 | 1.38±0.15 | 1.28±0.19 | 1.24±0.14 | 1.00±0.11 | 0.90±0.10 |

Note: All data are presented as mean±standard deviation (SD, n=3).
